# Outcomes of Living-Donor Liver Transplantation for Cholangiocarcinoma Versus Hepatocellular Carcinoma in the United States

**DOI:** 10.3390/jcm14207306

**Published:** 2025-10-16

**Authors:** Merve Gurakar, Ozge Sila Ozgur, Ayse Eylul Donmez, Nazli Begum Ozturk, Mehmet M. Bilgili, Ximena Parraga, Marwan Alsaqa, Eyad Abdulrazzak, Shanmukh Sai Pavan Lingamsetty, Alan Bonder, Ahmet Gurakar, Behnam Saberi

**Affiliations:** 1 Division of Gastroenterology and Hepatology, Johns Hopkins University School of Medicine, 720 Rutland Avenue, Ross Research Building, Suite 918, Baltimore, MD 21205, USA; mguraka1@jhmi.edu (M.G.);; 2 Department of Medicine, University of Pittsburgh Medical Center, Pittsburgh, PA 15213, USA; 3 Department of Radiology, Boston Children’s Hospital, Boston, MA 02115, USA; ayeyluldonmez@gmail.com; 4 Division of Gastroenterology and Hepatology, Saint Louis University School of Medicine, St. Louis, MO 63104, USA; 5 Division of Gastroenterology and Hepatology, Harvard Medical School, Beth Israel Deaconess Medical Center, Boston, MA 02215, USAmalsaqa@bidmc.harvard.edu (M.A.); abonder@bidmc.harvard.edu (A.B.); bsaberi@bidmc.harvard.edu (B.S.); 6 Department of Internal Medicine, Harvard Medical School, Beth Israel Deaconess Medical Center, Boston, MA 02215, USA

**Keywords:** cholangiocarcinoma, hepatocellular carcinoma, liver transplantation, living-donor liver transplantation

## Abstract

**Objectives:** Cholangiocarcinoma (CCA) is a malignancy of the biliary epithelium associated with high mortality. Hepatocellular carcinoma (HCC) is the most common primary liver tumor and has an established indication for liver transplant (LT). Living-donor LT (LDLT) may offer a curative and timely option for selected CCA patients. **Methods:** The United Network of Organ Sharing/Organ Procurement and Transplantation Network (UNOS/OPTN) data was reviewed from January 2010 to December 2022 for patients who underwent LDLT with HCC or CCA diagnostic codes. Post-LDLT survival for CCA was compared to HCC and non-HCC/CCA patients using Kaplan–Meier analysis. Stepwise multivariate analysis adjusted for recipient age, gender, race, and underlying etiology. **Results:** LDLT for CCA was most commonly performed in UNOS region 7 (54.9%) and region 2 (12.7%). Among 3993 LDLT recipients, 102 (2.6%) had CCA, 774 (19.4%) had HCC, and 3117 (78%) had neither HCC nor CCA. The 1-, 3- and 5-year post-LDLT survival of patients with CCA were 84.6%, 70.4%, and 62.2% versus 93.1%, 85.2%, and 78.2% for HCC, respectively. **Conclusions:** CCA accounts for 2.6% of LDLTs compared to 19.4% for HCC in the United States. At 5 years post-LDLT, patients with CCA have significantly lower survival compared to HCC.

## 1. Introduction

Cholangiocarcinoma (CCA) is an aggressive malignancy arising from the biliary epithelium and it is also the second most common primary liver malignancy after hepatocellular carcinoma (HCC) [1,2,3]. CCAs are traditionally classified into three subtypes depending on their anatomical site of origin: intrahepatic (iCCA), perihilar (pCCA), and distal (dCCA) CCA [1,4]. Among these, perihilar CCA is the most common type and accounts for approximately 50–60% of all CCA cases [5]. Although CCA is rare, occurring in less than 6 persons per 100,000 population [1], it is associated with a poor prognosis, mainly due to the delayed diagnosis, limited treatment options, and aggressive disease course, with a 5-year overall survival rate of less than 10% [6,7,8] and a high rate (>75%) of recurrence [9,10]. Radical surgical resection with clear margins can offer a potential cure for patients with resectable tumors. However, this option is often not feasible due to locally advanced or metastatic disease at the time of presentation [11,12].

Liver transplant (LT) allows for a radical and potentially curative treatment option for patients with locally advanced iCCA and pCCA without distant metastases [13,14]. Early results for LT alone for CCA were uniformly poor, largely due to a high incidence of recurrence (~50%) and mortality (5-year survival rate of only about ~17%) [15]. A protocol for neoadjuvant chemoradiation for unresectable pCCA was developed by the Mayo Clinic in 1993 [16]. The Mayo Clinic protocol emphasizes rigorous selection of patients with early-stage CCA, specifically those whose tumors are considered unresectable or that develop in the context of an underlying primary sclerosing cholangitis (PSC) [17]. Eligible tumors must measure less than 3 cm in maximal radial diameter, and patients must show no signs of intrahepatic or extrahepatic metastases. Neoadjuvant chemoradiation therapy includes 2 weeks of external beam therapy with 5-fluorouracil infusion, followed by transcatheter brachytherapy, and is then continued with maintenance oral capecitabine [18,19]. Although the Mayo protocol includes brachytherapy, the group recognizes that brachytherapy is technically difficult and resource intensive. In their analysis, they conclude that there is no added benefit in giving brachytherapy compared with external beam radiotherapy alone [20]. Staging abdominal exploration is performed prior to transplantation to exclude regional lymph node metastases, peritoneal metastases, or locally extensive disease.

Preliminary pilot study results for 11 patients undergoing liver transplantation for unresectable CCA under the Mayo Clinic protocol were reported in the year 2000 and were encouraging [21]. A few years later in 2004, Heimbach et al. reported 82% 5-year survival for 28 patients with unresectable pCCA after LT [22]. Rea et al. examined Mayo Clinic data from 1993 to 2004. They compared 38 patients receiving neoadjuvant therapy with LT for unresectable pCCA to patients who underwent local resection. LT had superior outcomes, with 5-year survival for LT vs. resection of 82% vs. 21%, respectively [16]. These data not only confirmed the feasibility of the protocol but also highlighted the critical role of neoadjuvant therapy in optimizing outcomes. Follow-up data from 2006 showed that in 65 patients undergoing neoadjuvant therapy followed by deceased-donor or living-donor LT, 1- and 5-year survival were 91% and 76%, similar to other more established indications for liver transplantation, such as hepatitis-C-virus-related cirrhosis and HCC [23]. This led to the increased acceptance of LT as a feasible treatment option, gradually solidifying its role and establishing it as the standard of care in several highly experienced centers in the US [24].

Living-donor liver transplant (LDLT) offers unique advantages over deceased-donor LT in patients with pCCA. In LDLT, (1) the operation is easily schedulable and can be planned in advance without significant delay, (2) chemotherapy can be interrupted and carefully resumed in a controlled manner according to the planned operation schedule, and (3) the pool of donor organs for “standard indications” is not burdened, thus minimizing the demand of organs from deceased donors and optimizing the organ allocation efficiency [25]. LDLT with neoadjuvant chemoradiation has been utilized to manage unresectable pCCA in highly selected patients, though data on post-LDLT survival are limited.

Based on this prior work, the present study aimed (1) to evaluate the trend of LDLT in CCA patients in the US using the United Network for Organ Sharing (UNOS) and Organ Procurement and Transplantation Network (OPTN) database, (2) to compare survival rate in patients with CCA and HCC post-LDLT, and (3) to evaluate the predictors of mortality in CCA post-LDLT, in order to provide more comprehensive insight into factors influencing outcomes in this complex patient population.

## 2. Materials and Methods

### 2.1. Data Source

We used the United Network of Organ Sharing (UNOS) database, which serves as a non-profit Organ Procurement and Transplantation Network (OPTN) contractor that collaborates with the federal government to administrate the transplant system in the US. Institutional Review Board approval was not required for this study as the database is de-identified and publicly available. The reported interpretations and conclusions in this article are the concern of the authors only and should not be supposed official opinions of the OPTN or the US government.

### 2.2. Study Design and Patient Population

Data collected included adult patients > 18 years old, diagnosed with CCA and bile duct cancer (diagnostic codes 4403 and 4420), who underwent LDLT from January 2010 to December 2022. Diagnosis of CCA was coded by the listing transplant center, generally based on pretransplant clinical, imaging, and cytologic findings. Explant pathology data were not available in the UNOS database. Patients with mixed HCC/CCA were excluded from the study. Data on recipient gender, age, race/ethnicity, and clinical data such as the model for end-stage liver disease (MELD) score, diagnosis of primary sclerosing cholangitis (PSC), diabetes mellitus, ascites, hepatic encephalopathy, presence of transjugular intrahepatic portosystemic shunt (TIPS) at the time of the transplantation, and the donor information including age and gender were collected.

### 2.3. Study Outcome

The primary outcome of the study was post-LDLT survival. Survival probabilities were estimated with Kaplan–Meier methods, and multivariable stepwise analyses were conducted with adjustments for donor and recipient characteristics.

### 2.4. Statistical Methods

We performed all statistical analyses using Stata software, version 16.1 (StataCorp LLC, College Station, TX, USA). The categorical variables were reported as numbers with percentages, and the continuous variables were reported as means with standard deviations. Categorical variables were compared using the chi-square test, whereas continuous variables were analyzed with the t-test; corresponding *p*-values were reported. Survival outcomes for the two groups were evaluated with Kaplan–Meier estimates, and differences between survival curves were assessed using the log-rank test. To identify independent predictors of survival, we carried out forward stepwise multivariable Cox regression analyses, adjusting for both donor- and recipient-related factors. Entry into the model was set at a significance level of 0.05, while variables were removed if the *p*-value exceeded 0.10. Clinically relevant factors along with those that demonstrated statistical significance in univariate analysis were considered for inclusion. Covariates entered into the model included age, sex, race, primary sclerosing cholangitis (PSC), MELD score, serum creatinine, diabetes, ascites, hepatic encephalopathy, prior TIPS, donor age, and donor sex. Hazard ratios (HRs) were reported with 95% confidence intervals (CIs). Statistical significance was determined at a threshold of *p* < 0.05.

## 3. Results

### 3.1. Baseline Characteristics of LDLT Recipients with CCA Versus HCC

**Figure 1:**
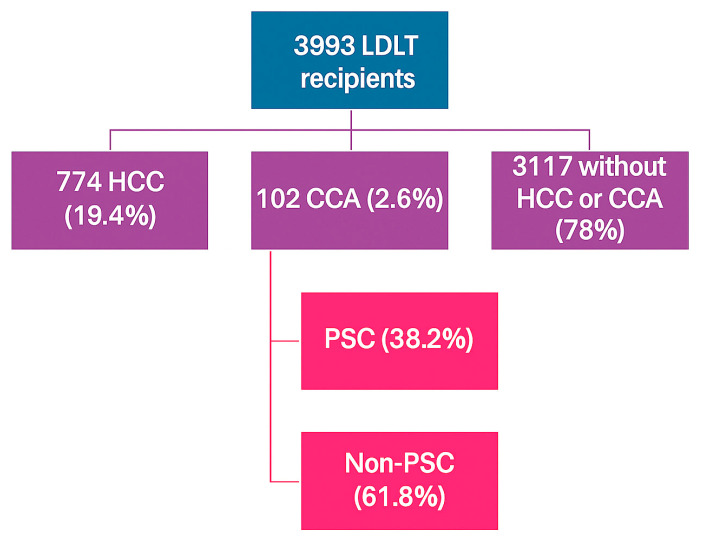
Distribution of LDLT recipients.

A total of 3993 LDLT recipients were identified. Among these patients, 774 (19.4%) had HCC, and 102 (2.6%) had CCA (Figure 1). Table 1 summarizes the demographics and clinical characteristics of the study cohort. Patients with CCA were younger (51.0 +/− 11.9 years vs. 59.1 +/− 9.9 years, *p* < 0.001) and more likely to be Caucasian (96% vs. 71.4%, *p* < 0.001) than patients with HCC. Gender was comparable between the groups. There were 39 (38.2%) patients with PSC-related CCA. Patients with CCA were more likely to have concurrent PSC diagnosis compared to patients with HCC (38.2% vs. 3.2%, *p* < 0.001). The MELD scores prior to LT were similar between both groups (12.0 mean vs. 13.9 mean, *p* = 0.66). Patients with CCA were less likely to have diabetes (8.8% vs. 29.4%), ascites (20.5% vs. 53.3%), hepatic encephalopathy (8.8% vs. 41.0%), and TIPS (1.0% vs. 8.9%) than patients with HCC (*p* < 0.001). Among patients with HCC, there were 354 (45.7%) patients with hepatitis C virus infection, 74 (9.5%) with alcohol-related liver disease, 158 (20.4%) with metabolic dysfunction-associated steatotic liver disease (MASLD), 42 (5.4%) with hepatitis B virus infection, and 25 (3.2%) with PSC (Supplemental Table S1).

### 3.2. Percentage of LDLT by Indication and UNOS Region

LDLT for CCA was most commonly performed in two UNOS regions—region 7 (54.9%) and region 2 (12.7%)—while LDLT for HCC was performed across three UNOS regions—region 2 (20.9%), region 5 (18%), and region 9 (15.9%).

### 3.3. Survival Analysis Between CCA Versus HCC Patients After LDLT

**Figure 2:**
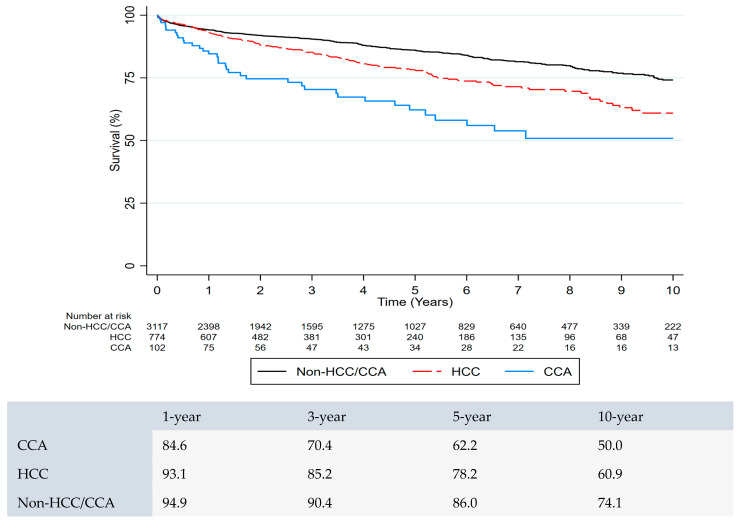
Survival comparison between HCC and CCA patients following living donor liver transplant.

The 1-, 3-, and 5-year post-LDLT survival rates of CCA patients (*n* = 102) were 84.6%, 70.4%, and 62.2%; the survival rates of HCC patients (*n* = 774) were 93.1%, 85.2%, and 78.2%, respectively. Overall, 1-, 3-, and 5-year survival rates were significantly lower in CCA patients compared to HCC patients after LDLT (*p* < 0.001). In multivariate analysis, among the patients who underwent LDLT, the CCA group was associated with higher mortality than the HCC and non-HCC/CCA groups (Figure 2).

In multivariable analysis, predictors of mortality after LDLT were age (HR = 1.24 [1.15–1.34], *p* < 0.001), HCC (HR = 1.45 [1.11–1.62], *p* = 0.002), CCA (HR = 2.98 [2.12–4.18], *p* < 0.001), creatinine (HR = 1.13, [1.16–1.43], *p* < 0.001), and diabetes (HR = 1.31 [1.31–1.10], *p* = 0.003) (Table 2).

**Figure 3:**
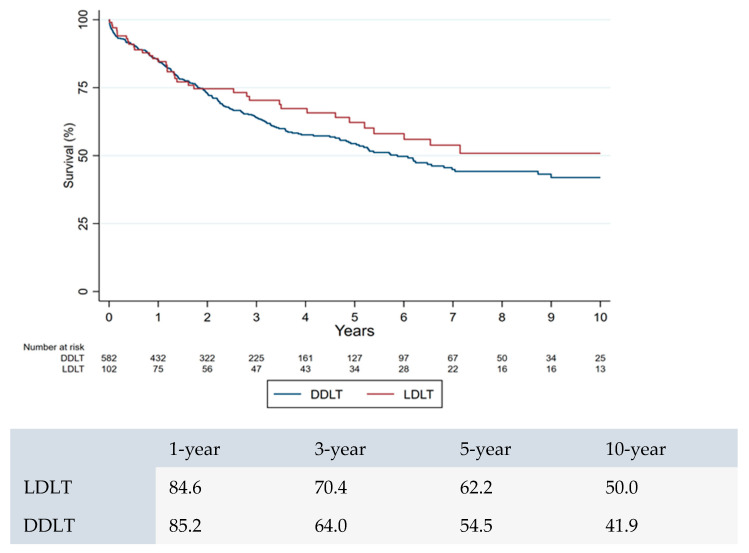
LDLT vs. DLDT survival in patients with CCA.

There was no significant difference in 1-, 3-, and 5-year survival rates for CCA after DDLT and LDLT (*p* = 0.24) (Figure 3).

## 4. Discussion

Although rare, CCA represents a growing clinical challenge due to the highly aggressive nature of disease behavior and, more importantly, its tendency for late presentation [11,12]. The high recurrence rate following treatment [9,10] and poor long-term survival [6,7,8] further underscore and emphasize the urgent need for improved therapeutic strategies. Conventional surgical approaches are frequently limited by tumor location, underlying liver disease, or patient comorbidities, ultimately leaving many patients with very few truly effective therapeutic options available for curative treatment. These challenges have prompted exploration of alternative approaches, including liver transplantation in carefully selected cases.

Historically, LT for treatment of CCA was limited due to both poor long-term outcomes and high recurrence rates [15,26,27], as well as the limited availability of donor organs. For patients with pCCA, the development of protocols that include neoadjuvant chemoradiotherapy followed by LT has yielded acceptable outcomes compared to LT alone [15,16,21,22,23,28,29,30]. Currently, the standard MELD exception score for pCCA, set by UNOS, is at the Median MELD at transplant (MMaT) minus 3 points [31]. Patients must have unresectable disease, which can be due to either a locally advanced tumor with significant vascular and/or biliary invasion that prevents complete resection or, alternatively, a compromised hepatic functional reserve due to underlying liver disease. The patient must have a single tumor less than 3 cm in radial diameter with no evidence of intra- or extrahepatic metastasis and have undergone neoadjuvant chemoradiation therapy at a center with an approved protocol. Given these constraints and the lengthy waitlist for LT, LDLT offers a timely alternative, eventually minimizing the risk of deceased-donor-transplant-waitlist-associated morbidity and mortality for these high-risk patients [25,32].

We showed that LDLT for the management of unresectable CCA is available in certain regions (UNOS region 7, 55% of the cases; UNOS region 2, 12.7% of the cases) of the US with expertise under strict selection protocols. This regional concentration reflects the presence of experienced, high-volume transplant programs in the Midwest and Midatlantic that have established rigorous protocols requiring unresectable but non-metastatic disease, radiographic stability during neoadjuvant therapy, and operative staging to exclude extrahepatic spread [17,18,19]. Post-LDLT outcomes depend on patient selection and adherence to these protocols, given the substantial risk of post-LT recurrence and mortality [10]. In our cohort, survival was significantly lower among CCA recipients compared with HCC recipients, with 5-year post-LDLT survival of 62.2% versus 78%. Despite a lower survival rate, LDLT remains an acceptable option for curative therapy with a 5-year survival rate > 50% [33]. The limited distribution of LDLT for CCA across the remainder of the UNOS regions suggests that broader dissemination of standardized protocols and training will be necessary to expand access to this potentially curative option. Center effects on post-transplant outcomes in patients with CCA should also be recognized when developing CCA protocols, as patient survival in LT recipients with pCCA in less-experienced centers has consistently been shown to be worse than that observed in well-experienced centers [34].

Intrahepatic CCA (iCCA) as an indication of LT remains controversial given initial disappointing and discouraging clinical results [35,36]. Recent analyses suggest that LT may be a potentially feasible option for highly selected patients with favorable tumor biology [37,38]. If disease stability can be demonstrated for at least 6 months on neoadjuvant therapy without extrahepatic disease, LT can be considered for iCCA under investigational protocols [39,40]. There are two recent prospective case series of patients from Houston Methodist Hospital with unresectable iCCA managed with LT and neoadjuvant chemotherapy [41,42]. In both series, roughly one third of patients referred and one half of patients listed ultimately underwent LT. Overall survival at 1, 3, and 5 years was 100%, 83%, and 83%, respectively, in the first study [41] and 100%, 71%, and 57%, respectively, in the second study [42]. LDLT offers a valuable solution for iCCA treatment, since it allows patients to avoid the prolonged waiting time on the list and higher dropout rates that are commonly observed in iCCA candidates for deceased-donor LT. In the US, the University of Pittsburgh [43] also has an established iCCA LDLT protocol. While the literature on this topic remains limited [44], there is at least one additional prospective trial that is expected to further define the role of LDLT in iCCA [45]. Further studies are needed to identify LDLT criteria and factors predicting survival.

There are several limitations in this study. Firstly, the survival comparisons between CCA and HCC are limited by the smaller sample size of the CCA cohort, which naturally restricts the statistical power of the analysis and reduces the ability to draw firm or broadly generalizable conclusions. Secondly, disease diagnosis was based on UNOS codes. Most CCA patients did not have pathological confirmation of disease (which is not available in the UNOS database). It is difficult to obtain pathology confirmation of CCA by biopsy or brush cytology. The diagnosis relies on intraluminal brushings and biliary aspiration for examination by fluorescence in situ hybridization (FISH), the elevation of carbohydrate antigen 19-9 (CA 19-9), and the presence of a mass on cross-sectional imaging in the presence of a malignant-appearing biliary stricture [33]. Endoscopic transgastric or percutaneous transhepatic biopsies of the tumor to establish diagnosis were excluded in the Mayo protocol, primarily because of the significant concern regarding the risk of seeding of metastases in the peritoneum, which would ultimately compromise transplant eligibility [46]. We were not able to differentiate between intrahepatic, perihilar, and distal CCA subtypes because the UNOS registry does not provide this level of diagnostic granularity, thus preventing more nuanced subgroup analyses that could have enriched the study findings. Other oncologic details regarding tumor size, tumor markers, tumor differentiation, and pretransplant treatment history were also not available in the UNOS registry. Lastly, given that LDLT for CCA was concentrated in a limited number of centers, generalizability may be limited across all regions.

## 5. Conclusions

In conclusion, LDLT with neoadjuvant chemoradiation has emerged as a feasible treatment option for unresectable CCA in carefully selected patients. Under strict selection criteria and guidelines, outcomes comparable or superior to surgical resection have been achieved. LDLT for pCCA has also achieved outcome results comparable to other indications for LT, thereby justifying both MELD score exception and the potential, carefully considered use of living-donor organs. The evidence for LDLT use among iCCA cases remains limited, but some studies have shown promise in patients with early-stage iCCA, comparable or even superior to resection. Continued advancements in LDLT techniques, postoperative care, and the development of new chemotherapeutic and biological agents may further enhance outcomes in patients undergoing LDLT for this indication. Additionally, continued refinement of patient selection criteria will be helpful to improve the outcomes among patients who undergo LDLT and exclude those who are at high risk of falling out of the preset criteria, prior to transplantation. Larger and higher-quality studies are essential to better define the role of LDLT for each tumor subtype, ultimately guiding clinicians toward more standardized and evidence-based approaches in this evolving field.

## Figures and Tables

**Table 1:** Demographics and clinical characteristics of the study cohort.

	CCA (*n* = 102)	HCC (*n* = 774)	*p*-Value
Age, years (mean, SD)	51.0 (11.9)	59.1 (9.9)	<0.001
Gender, Male (*n*, %)	70 (68.6%)	507 (65.5%)	0.53
Race			<0.001
White	98.1 (96%)	553 (71.4%)	
Black	1 (0.9%)	30 (3.8%)	
Hispanic	2 (1.9%)	132 (17.0%)	
Asian	0 (0.0%)	53 (6.8%)	
Other	1 (0.9%)	6 (0.7%)	
PSC	39 (38.2%)	25 (3.2%)	<0.001
MELD (Mean, SD)	12.0 (6.0)	13.9 (5.8)	0.66
Diabetes	9 (8.8%)	228 (29.4%)	<0.001
Ascites	21 (20.5%)	413 (53.3%)	<0.001
Hepatic encephalopathy	9 (8.8%)	318 (41.0%)	<0.001
TIPS	1 (1.0%)	69 (8.9%)	<0.001
Donor Age	37.3 (10.3)	38.5 (11.6)	0.30
Donor gender, Male	54 (52.9%)	359 (46.3%)	0.21

**Table 2:** Predictors of mortality in LDLT recipients.

	Hazard Ratio	95% Confidence Interval		*p* Value
Age	1.238	1.146	1.337	<0.001
Non-HCC/CCA	Reference			
HCC	1.345	1.113	1.624	0.002
CCA	2.978	2.123	4.177	<0.001
Creatinine	1.1291	1.163	1.432	<0.001
Diabetes	1.306	1.092	1.562	0.003

## Data Availability

The data presented in this study are openly available in the United Network of Organ Sharing (UNOS) database.

## Supplementary Materials

The following supporting information can be downloaded at: https://www.mdpi.com/article/10.3390/jcm14207306/s1, Table S1: Etiology of the underlying liver disease among HCC and non-HCC/CCA patients undergoing living donor liver transplant. Figure S1: Trend of HCC and CCA in LDLT in US per Year.

## Abbreviations

ALD	Alcohol-Associated Liver Disease
CA 19-9	Carbohydrate Antigen 19-9
CCA	Cholangiocarcinoma
dCCA	Distal Cholangiocarcinoma
iCCA	Intrahepatic Cholangiocarcinoma
pCCA	Perihilar Cholangiocarcinoma
FISH	Fluorescence In Situ Hybridization
HCC	Hepatocellular Carcinoma
LT	Liver Transplant
LDLT	Living-Donor Liver Transplant
MASLD	Metabolic-Dysfunction-Associated Steatotic Liver Disease
OPTN	Organ Procurement and Transplantation Network
PSC	Primary Sclerosing Cholangitis
TIPS	Transjugular Intrahepatic Portosystemic Shunt
UNOS	United Network for Organ Sharing
US	United States
