# Supplementary material for: Outcomes of Living-Donor Liver Transplantation for Cholangiocarcinoma Versus Hepatocellular Carcinoma in the United States
Source: J Clin Med. 2025 Oct 16;14(20):7306. doi: 10.3390/jcm14207306 (PMC12565021; doi:10.3390/jcm14207306)
Supplement: Supplementary file 1 [file jcm-14-07306-s001.zip › jcm-3765869-supplementary.pdf]

Supplemental

Table S1. Etiology of the underlying liver disease among HCC and non-HCC/CCA patients undergoing living donor liver transplant

	**HCC (n=774)**	**Non-HCC/CCA (n=3117)**
HCV	354 (45.7%)	367 (11.8%)
ALD	74 (9.5%)	505 (16.2%)
MASLD	158 (20.4%)	668 (21.4%)
HBV	42 (5.4%)	36 (1.1%)
PSC	25 (3.2%)	655 (21.0%)
Other	146 (18.8)	886 (28.4%)

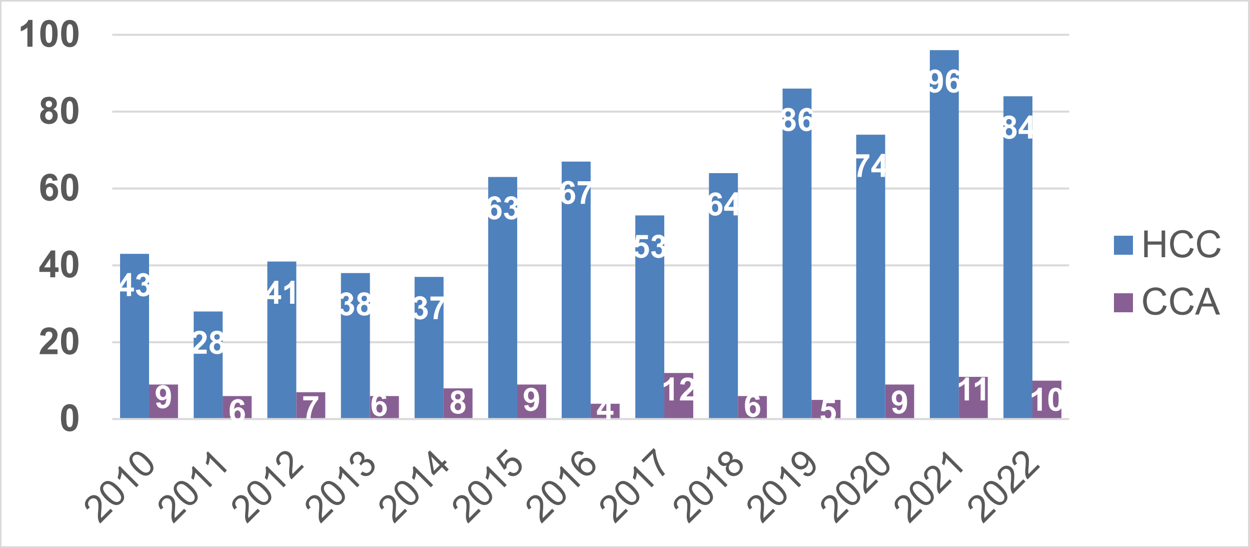

Figure S1. Trend of HCC and CCA in LDLT in US per Year.
